# Medical Jurisprudence

**Published:** 1829

**Authors:** 


Art. VII.—Medical Jurisprudence. *Sequel of the case of John Birdsell, with remarks on Feigned Insanity.* By the Editor.

To such as may have read in the last number of this Journal, the report of the trial of John Birdsell, some notice of what followed his conviction, may not be uninteresting. From the period of that event, to the day appointed for his execution, he remained in jail; at no time attempting, nor, apparently, meditating an escape. By a statute of Ohio, the Governor has the power of commuting the punishment by death, for perpetual confinement in the cells of the penitentiary, the convict subscribing a written assent to the exchange. A week before the day for his execution, several citizens of Cincinnati,—impressed with the opinion that he murdered his wife when insane, and, therefore, that his execution would be a violation of the law; but, at the same time, considering that his liberation might be dangerous to society,—conceived the design of procuring a commutation of his punishment. Accordingly, they circulated a petition to the Executive, praying for the same, and obtained a respectable number of signatures; among whom were ten or twelve physicians, and twice that number of advocates, all of whom had made themselves acquainted with the facts relating to his state of mind, at the time he destroyed his wife. Along with this petition they sent a statement of the testimony. His Excellency granted their request, and the culprit is now in the penitentiary.

It will be recollected by those who have read the report of his trial, that after Birdsell was committed to prison, his insanity ceased. When arraigned, he admitted that he had killed his wife; but insisted, that undoubtedly he was deranged, for he never intended to commit such an act. In this state of mind, he continued for some time after his conviction, when he again began to show symptoms of derangement. Being told of this change, I had the curiosity to visit him. He conversed rationally on every subject, till I touched upon the cause of his condemnation and imprisonment, when he declared that he had *not* killed his wife, and that she was still alive. I told him the evidences of the fact, were too strong to be resisted; to which he replied, that a *combination* of persons had sworn falsely against him; that, if he had ever admitted that he murdered her, he was now convinced that he was mistaken; for she had lately, not only spoken to him through the walls of the jail, but actually visited his apartment several times. When I inquired why he did not detain her, he answered that she came in with other women, and there was always something in her appearance, that made him *doubt* at the time, whether it was she; but that after her departure, and he reflected on what he had seen, he became convinced that she was alive, and had been with him. However, he placed still greater reliance on the sense of hearing. This certainly had not deceived him; and he had heard her speak almost every night for two or three weeks. She had been in the next room, opposite the head of his bed, and had conversed with him through the wall, on various subjects. In attempting to reason with him on the absurdity of denying that she was dead, he either felt or feigned a great deal of anger, and spoke violently and incoherently of those who had conspired against him.

Next day, I revisited, and found him tranquil. He conversed, temperately and rationally, on every subject, and made no allusion to his wife or his crime. When I started the subject, he went over a part of what he had contended for the day before; but was milder, less confident of the truth of his late observations, and in short, manifested much less insanity.

At my third visit, I found him rather more excited. On this occasion, while he was ignorant that a commutation of punishment had been granted, and when little more than a day would bring round the time for his execution, I proposed to him, in presence of the sheriff, the clerk of the court, one of his advocates, and some other gentlemen, to petition the Governor for an unconditional pardon; inserting in his petition, an admission of the fact, that he had killed his wife; but alleging that he must have been insane, or he could not have done so. The draft of a petition to this effect, was then read to him, by one of the gentlemen present. He rejected it with violence, declaring that it contained a lie, for which his wife would confront him at the bar of God, and for which his soul would be sent to hell. The gentlemen present urged him; proposing to forward with the petition, by a special messenger, the proofs of his insanity, at the time he committed the murder; and they expressed their belief, that a reprieve could thus be obtained. On pressing him, however, he manifested equal temper and obstinacy; seemed disposed to quarrel with all around him, asserted that his wife was not dead, and declared that he would sooner die, than subscribe his name to such a falsehood. Of all the gentlemen present, there was not one, as I assured myself at the time, who, from the countenance, manner, and words of the culprit, to say nothing of the *absurdity* of his refusal, could resist the conviction, that he was sincere. I need scarcely say, that the proposition was designed to test the sincerity of his recent declarations that he believed his wife to be alive; but he could have known nothing of this design, as it was communicated to none, but one of his counsel.

Let us inquire into the conclusion which should be drawn from the facts which have been detailed.

They prove one of two things—that a partial insanity had returned upon him, or that he was feigning it. Which of these theories will best explain the facts? I cannot hesitate to choose the former.

1. It appears from the testimony on the trial, that Birdsell's propensity to drink, the indulgence of which excited *mania a potu*, was periodical, and returned four or five times a year. He was delirious and killed his wife early in March, soon after which, as in previous times, the delirium went off; but, in July, four months afterwards, he exhibited symptoms of a mild monomania.

Now I would submit to the profession, whether the state of his nervous system, which attended the other paroxysms, might not, and would not, probably, recur in a partial degree, when the usual period arrived? His periodical drunkenness, and subsequent mania, could not be ascribed to periodical temptation, for his opportunities and the enticements to drink were always the same; and if not attributable to these, they could only have arisen from the recurrence, at distant periods, of a peculiar state of the nervous system. The debilitating circumstances of low diet, darkness, silence, and damp walls, were, moreover, well calculated to reproduce a disease, to the cure of which they are well known to be unfavorable.

2. At two of my visits, when the culprit was at perfect rest, and unexcited by conversation, I found his pulse from 86 to 94 beats in a minute. I am aware, from observation, that in assigning an unnatural frequency of the pulse as an invariable concomitant of insanity, Dr. Rush has fallen into an error; for that state of the mind may co-exist with a natural pulse. Still, when a frequent pulse is present in cases of a dubious character, its value must be admitted; for it cannot like incoherent conversation, depend on the will of the individual.

While on this subject, as Dr. Rush's authority is deservedly great, in the United States, I will take occasion to add, that I have seen many cases of mania, monomania, and hypochondriasm, in which the pulse was not accelerated, and some in which it was unnaturally slow. Eighteen months ago I several times saw a patient with Dr. Marshall, of this city, a youth, who labored under mania, and had a pulse which, for the greater part of the time, rose very little above the natural standard; and at presest I am attending a young gentleman from Virginia, whose pulse is, habitually between 60 and 70, while his loquacity is incessant, and his locomotion so untiring and disorderly, as to require the restraint of a straight waistcoast.

3. It has been said that frequent spitting is a sign of impending madness. Of this I have myself known two examples. In my visits to Birdsell, I observed, although he was not a tobacco chewer, that he spit incessantly.

4. A disordered state of the senses of seeing and hearing, was a predominant circumstance in the delirium of Birdsell when he killed his wife, and in all the preceding attacks. Now the delirium which he exhibited in jail, was precisely of this kind. He thought that he saw his wife, he often heard her talk, and she even conversed with him on ordinary topics through the wall.

5. In his previous attacks, his derangement was, as far as we know, generally confined to one subject, and his wife was the principal person involved in it. I need scarcely remind the reader that this was the character of the delirium which he manifested in prison.

6. If Birdsell had feigned the delirium into which we are inquiring, he would not have restricted himself to such narrow limits, nor been so reserved in the display of his maniacal ideas. Above all, he would not have ventured to abate in its manifestations, to the degree which I witnessed on my second visit, as already related. He would have kept it up with increasing violence.

7. It is absurd to suppose insanity feigned without a motive. But what could have moved Birdsell to practice such an imposition? His trial was over and sentence of death had been passed upon him. After his apprehension, and when his defence was about to be rested on mental derangement, he did not attempt to simulate that condition of mind; but appeared quite rational, though it might, then, have been of advantage to him to counterfeit madness. It is not probable, then, that at a subsequent period, when he could in no way be benefitted by such a trick, he would have undertaken to practice it.

8. If his derangement had been pretended, why would he have refused to sign a petition for pardon, because it contained an admission that he had killed his wife; when he had repeatedly acknowledged the fact before the trial, and, if of sound mind, must have known that it had been put beyond all dispute? The proposed application for an unconditional reprieve, was not based upon his insanity *after* conviction, but at the time of the murder. He wished to live and was willing to petition the Governor; but when we proposed to introduce into the petition, a confession that he had murdered his wife in a fit of derangement, he refused with obstinacy and ill temper, because it came athwart his maniacal idea.

9. If Birdsell had refused the commutation of his punishment and been hung, it would have been regarded by all who knew the fact, as a proof of insanity; as his subscribing to the exchange has been pronounced an evidence of sound mind. But the conclusion drawn from this acquiescence is entirely unwarrantable. I have already stated, that his prison hallucination consisted in the belief,—excited by the testimony of his disordered senses,—that his wife was still alive; and consequently, that the combination, which in former paroxysms, he had so much dreaded, had sworn falsely against him. Now all this might have existed, and yet his mind been regular on every other subject; and hence he might have balanced between the gallows and confinement for life, while he believed both of them unjust. To deny this, would be equivalent to denying the *existence* of monomania or partial insanity, the most common form of mental alienation. Had his consenting to the commutation turned upon the admission of his guilt, I have no doubt that he would have been hung.

From these different facts and considerations, I am convinced, that the prison delirium of Birdsell was not feigned, but real; but if it were the former, the conclusion attempted to be drawn from it—that he was not deranged when he killed his wife—is wholly unwarrantable. The proofs of his madness at that time are independent of what followed his condemnation; and can scarcely fail to carry conviction into the mind of any professional man, who will condescend to study them with seriousness.

To the fate of Birdsell—referring to himself only—I have always felt indifferent; but having been drawn, officially, to the study of his case, I have endeavored to make it of some value as a matter of Medical Jurisprudence; and am gratified to know, that what has been already published, has drawn attention, to a much neglected but deeply interesting subject. To the same end, I have written this sequel; which I hope will direct the inquiries of the younger members of the profession, to the subject of feigned insanity, concerning which the oldest physicians and jurists may often be perplexed. That Birdsell was a bad man, I have as little doubt, as that he killed his wife—not in a fit of drunkenness—but in a paroxysm of insanity. But a good man under a similar delusion might have done the same thing; and hence the importance of considering this last dreadful act of his social life on its own merits, and disconnecting it from his previous conduct. This should only have been referred to, in the absence of proof that he had committed the murder. When society shall come to punish a *special* act in one man and excuse it in another, its jurisprudence will no longer rest upon those principles of justice, which all are interested in maintaining. The law should not be allowed to cast its sword into the balance.

